# Drinking and Water Handling in the Medaka Intestine: A Possible Role of Claudin-15 in Paracellular Absorption?

**DOI:** 10.3390/ijms21051853

**Published:** 2020-03-08

**Authors:** Christian K. Tipsmark, Andreas M. Nielsen, Maryline C. Bossus, Laura V. Ellis, Christina Baun, Thomas L. Andersen, Jes Dreier, Jonathan R. Brewer, Steffen S. Madsen

**Affiliations:** 1 Department of Biological Sciences, University of Arkansas, SCEN 601, Fayetteville, AR 72701, USA; maryline.bossus@lyon.edu (M.C.B.); lvellis@email.uark.edu (L.V.E.); steffen@biology.sdu.dk (S.S.M.); 2 Department of Biology, University of Southern Denmark, Campusvej 55, 5230 Odense M, Denmark; Amorck@live.dk; 3 Department of Math and Sciences, Lyon College, 2300 Highland Rd, Batesville, AR 72501, USA; 4 Department of Nuclear Medicine, Odense University Hospital, Sdr. Boulevard 29, 5000 Odense C, Denmark; Christina.Baun@rsyd.dk (C.B.); Thomas.Andersen@rsyd.dk (T.L.A.); 5 Department of Biochemistry and Molecular Biology, University of Southern Denmark, Campusvej 55, 5230 Odense M, Denmark; jes.dreier@cpr.ku.dk (J.D.); brewer@memphys.sdu.dk (J.R.B.)

**Keywords:** aquaporin, claudin, drinking rate, epithelial fluid transport, enterocyte, occludin, osmoregulation, paracellular

## Abstract

When euryhaline fish move between fresh water (FW) and seawater (SW), the intestine undergoes functional changes to handle imbibed SW. In Japanese medaka, the potential transcellular aquaporin-mediated conduits for water are paradoxically downregulated during SW acclimation, suggesting paracellular transport to be of principal importance in hyperosmotic conditions. In mammals, intestinal claudin-15 (CLDN15) forms paracellular channels for small cations and water, which may participate in water transport. Since two *cldn15* paralogs, *cldn15a* and *cldn15b*, have previously been identified in medaka, we examined the salinity effects on their mRNA expression and immunolocalization in the intestine. In addition, we analyzed the drinking rate and intestinal water handling by adding non-absorbable radiotracers, 51-Cr-EDTA or 99-Tc-DTPA, to the water. The drinking rate was >2-fold higher in SW than FW-acclimated fish, and radiotracer experiments showed anterior accumulation in FW and posterior buildup in SW intestines. Salinity had no effect on expression of *cldn15a*, while *cldn15b* was approximately 100-fold higher in FW than SW. Despite differences in transcript dynamics, Cldn15a and Cldn15b proteins were both similarly localized in the apical tight junctions of enterocytes, co-localizing with occludin and with no apparent difference in localization and abundance between FW and SW. The stability of the Cldn15 protein suggests a physiological role in water transport in the medaka intestine.

## 1. Introduction

In fresh water (FW) fishes, the intestinal epithelium must limit excessive fluid absorption while securing dietary ion uptake [1]; in seawater (SW), imbibed water is absorbed in a solute-linked process [1,2]. Therefore, the functional plasticity of the enterocytic epithelium is a critical factor in euryhaline fish that are capable of going through salinity transitions. Elevated intestinal aquaporin (Aqp/*aqp*) abundance in eel [3,4,5] and salmonids [6] in response to SW transfer have led to propose a transcellular water path in these species [7]; however, in medaka, a consistent downregulation of several intestinal Aqp/*aqp* isoforms after SW transfer has challenged this model and suggests a major involvement of a paracellular pathway [8].

Transepithelial water transport has been suggested to be mainly transcellular via Aqps, but this matter is still under debate [9,10]. Thus, in leaky epithelia, similar to the intestine, fluid transport may primarily be paracellular as proposed based on a corneal model [9] or include both components as proposed for marine fish [2]. Taking species differences into account, it appears that the medaka intestine may be a choice comparative model to study paracellular fluid transport because a tight junction defined path seems central, as suggested by Madsen et al. [8].

Proteins belonging to the claudin (Cldn) superfamily are the main determinants of tight junction permeability properties and thus important regulators of paracellular transport [11,12]. Cldns are integral membrane proteins with 4 trans-membrane domains and two extracellular loops (ECL). The amino acid residues of the first ECL are critical for the permselectivity of the junction they create in homo- or hetero-dimeric and -tetrameric combinations [13,14,15,16]. There are 27 claudins (CLDNs) paralogs described in mammals [12]; in the teleost lineage, an extensive expansion of the *cldn* gene family due to gene duplications has led to a higher number with e.g., 56 in Fugu [17] and 54 in zebrafish [18]. The specific permselectivity has been investigated for several mammalian CLDN paralogs, and there are many examples of barrier-forming as well as specific anion- and cation-pore-forming CLDNs [12]. In addition, there are a few examples of CLDNs contributing to creating water-permeable pores. This has been convincingly demonstrated for CLDN2 [19], which has functional significance in the mammalian kidney, and most recently intestinal CLDN15 has also been assigned such a role [20] in addition to the cation-pore-forming properties of both CLDNs [12]. However, Na^+^ and water fluxes through CLDN15 inhibit each other in functional contrast to CLDN2 [20]. Based on amino acid homology, especially in the first ECL, it is often assumed that fish Cldns give rise to the same permeability properties as mammalian orthologues, but only a few have been investigated thoroughly [21,22]. Mutational analysis and MD simulations [15,16] based on the crystal structure [23] have shown that especially amino acid D55 is critical for CLDN15 pore formation. In support for a similar function in medaka to the mammalian orthologue is the conservation of this amino acid in both medaka Cldn15a and Cldn15b.

A given tissue often shows the expression of several CLDN paralogs [12]. In the mammalian nephron, this is coupled to a highly segmental pattern of expression [24]. In the mammalian intestine, CLDN15 appears to be one of the most abundant CLDNs, at least in the small intestine [25,26,27], and it plays a critical role in the gut ontogeny of both mammals and fish [28,29]. In mice, CLDN15-mediated Na^+^ back-flux into the intestinal lumen is essential for active glucose absorption through the Na^+^/glucose cotransporter, safeguarding monosaccharide uptake [30]. In fishes, Cldn15a paralogs have been found to be expressed specifically in the gastrointestinal (GI) tract (salmon [31,32], zebrafish [33], medaka [34]). In medaka, we previously identified an additional new paralog, Cldn15b, which is also primarily expressed in the intestine at levels several orders of magnitude higher than any other examined organs [34].

To develop our knowledge about paracellular versus transcellular fluid transport, it will be valuable to expand our understanding of water transport and enterocyte tight junctions in medaka. Furthermore, the functional plasticity of the intestine during salinity change in this euryhaline fish is useful when seeking to understand basic principles. Therefore, the goals of this work were to first study drinking behavior and water handling in response to changes in the osmotic environment, which are unknown in adult medaka. Secondly, we examined the expression and localization of the two Cldn15 paralogs in relation to hypo- to hyperosmotic acclimation based on the assumption that intestinal Cldn15 is implicated in water transport, as seen in other models.

## 2. Results

### 2.1. Drinking Rate and Intestinal Handling of Imbibed Water

**Figure 1:**
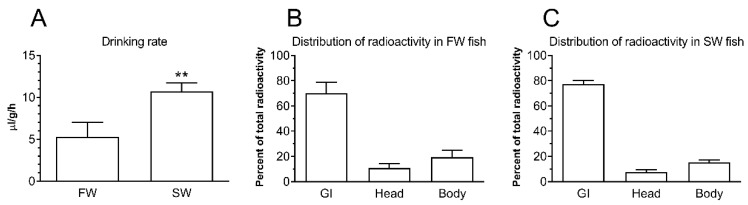
(**A**) Drinking rate (μL/g/h) in fresh water (FW) and seawater (SW)-acclimated medaka estimated by radioactivity in the entire gastrointestinal tract after incubation in 51-Cr-EDTA traced FW or SW for 3 h followed by rinsing in clean water for 1 h. ** *p* < 0.01. In (**B**) (FW) and (**C**) (SW), the radioactivity content of the gastrointestinal tract (GI, imbibed) is compared to radioactivity absorbed to the head and remaining body parts.

In preliminary experiments using both FW- and SW-acclimated fish, it was assured that the intestinal accumulation of radioactivity in fish continued linearly in excess of 3 h, and gut-passage time after drinking thus was well in excess of 3 h (data not shown). Therefore, the drinking rate estimation was based on a 3 h incubation in 51-Cr-EDTA containing water. The drinking rate was relatively high in FW-acclimated medaka (5 μL/g/h) but was doubled in fish acclimated to SW (Figure 1). Drinking rate measurements were based on counting radioactivity in the GI tract after incubation and a 1 h rinsing period in clean water.

The head and body carcass where counted separately after the experiment, and the radioactivity content in these parts amounted to 8–10% and 15–20%, respectively, of the total radioactivity of the fish (Figure 1B,C). It is assumed that this is mainly due to attachment to the external mucus layer in these body parts, as Cr-EDTA has been shown to be a non-absorbable marker, which means that it does not cross the intestinal epithelium [35].

**Figure 2:**
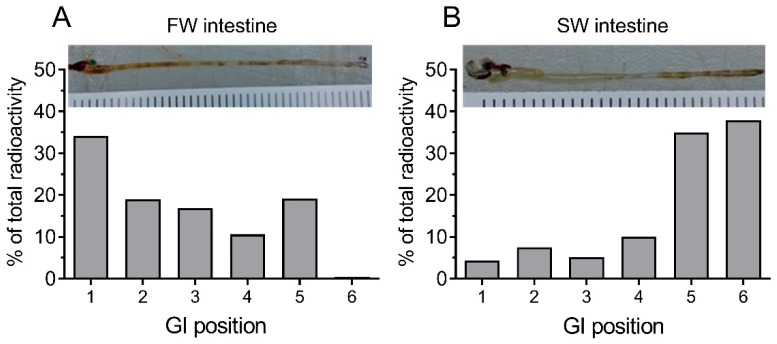
Distribution of radioactivity longitudinally in the gastrointestinal (GI) tract of fish allowed to drink 51-Cr-EDTA traced FW (**A**) or SW (**B**) water for 3 h. After incubation, the entire GI tract was ligatured into 0.5 cm segments and each segment was then transferred to a scintillation vial and scanned for radioactive content. The entire intestine was approximately 3 cm in length, as shown in the inserted photographs.

When dissected intestines were carefully fragmented into 0.5 cm segments and analyzed, it was found that radioactivity was evenly distributed but with a trend of showing higher levels at the anterior end of the FW intestine (Figure 2A). In the SW-acclimated fish, the radioactivity clearly accumulated toward the posterior end of the intestine (Figure 2B).

**Figure 3:**
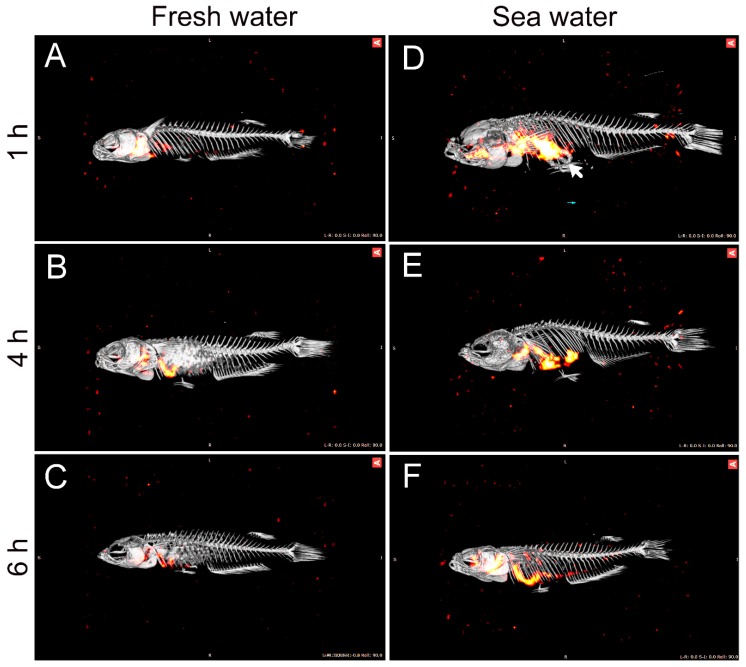
Visualization of the movement of imbibed water along the gastrointestinal tract of medaka acclimated to FW (**A**–**C**) or SW (**D**–**F**). Images are merged from single photon emission computed tomography (SPECT) (red intensity layer) and computed tomography (CT) (gray) scans of fish which had been incubated in 99-Tc-DTPA traced FW or SW for 1 h followed by transfer to non-radioactive FW or SW for 1 h (**A**, **D**), 4 h (**B**, **E**), or 6 h (**C**, **F**). The SPECT layer visualizes the localization (and intensity) of imbibed isotope-labeled water and the CT layer visualizes mineralized structures (skeleton and mineral precipitates in the SW intestines, white arrow in **D**). Note that 99-Tc has a short half-life (6 h), which influences the apparent intensities of the imbibed isotope. The fish was approximately 3 cm in length.

The progressive movement of imbibed water along the intestine was followed in a more direct way by a series of single photon emission computed tomography–computed tomography (SPECT-CT) scans of intact, euthanized fish after incubation in 99-Tc-DTPA-traced water (Figure 3). The imaging series showed an initial high intensity of tracer in the esophageal end of the GI tract with a more posterior distribution as the incubation time was increased. The precipitation of mineral salts (Mg- and Ca-carbonates) could be seen in the CT images of SW-acclimated fish ca 2/3 down the intestine (white arrow in Figure 3D).

### 2.2. Transcript Levels and Response to Salinity

**Figure 4:**
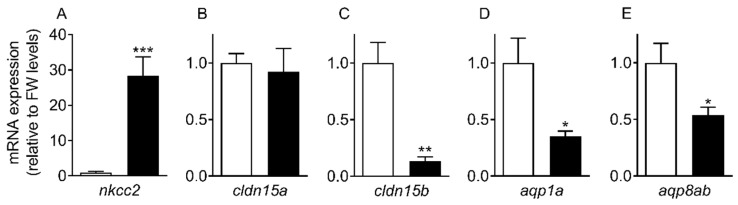
Transcript levels of *nkcc2* (**A**), *cldn15a* (**B**), *cldn15b* (**C**), *aqp1a* (**D**), and *aqp8ab* (**E**) in intestine from medaka acclimated to FW (white bars) or SW (black bars). Fish were acclimated to the respective salinities for over one month prior to sampling (*n* = 8). Expression levels represent the mean value ± SEM relative to FW levels. Asterisks indicate a significant difference from FW expression (* *p* < 0.05, ** *p* < 0.01, *** *p* < 0.001).

**Figure 5:**
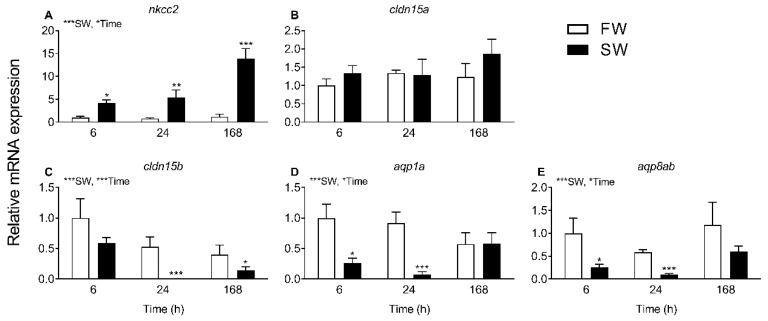
Effect of FW-to-SW transfer on intestinal transcript levels of *nkcc2* (**A**), *cldn15a* (**B**), *cldn15b* (**C**), *aqp1a* (**D**), and *aqp8ab* (**E**). Fish were transferred from FW-to-SW (black bars) or FW-to-FW (white bars) as a control and sampled at 6, 24, and 168 h (*n* = 6). Expression levels represent the mean value ± SEM relative to the 6 h-FW group. Asterisks next to SW and Time refers to the overall effects of a factor with two-way ANOVA. All targets with overall effects also had a significant interaction between factors, so the differences between time-matched groups were analyzed with Bonferroni multiple comparisons test (* *p* < 0.05, ** *p* < 0.01, *** *p* < 0.001) to identify the time-dependence of SW effects.

The transcript levels of selected targets were analyzed in intestines from medaka long-term acclimated to FW and SW (Figure 4). The absorptive Na^+^, K^+^,2Cl^−^ cotransporter (*nkcc2*) level was several-fold higher in SW than FW fish, whereas *cldn15b*, *aqp1a*, and *aqp8ab* levels were significantly reduced in SW compared to FW fish. *cldn15a* was unaffected by long-term salinity acclimation. The salinity-induced changes in transcript levels observed in long-term acclimated fish were reproduced in a 7-day time course experiment (Figure 5), with *nkcc2*, *cldn15b*, *aqp1a*, and *aqp8ab* all being significantly affected by both salinity and time (two-way ANOVA). Since there was a significant interaction between the two factors on these transcripts, the effect of SW was time-dependent. Thus, the SW effect on *nkcc2* was significant at all time-points, but it was the highest after 168 h. The effect on cldn15b was significant only after 24 h and 168 h days, while both *aqps* decreased already after 6 h and 24 h but not significantly so at the 168 h time point. *cldn15a* was unaffected by salinity during the 7-day time course experiment, as observed in long-term acclimated fish.

### 2.3. Cldn15 Localization in the Intestinal Epithelium

**Figure 6:**
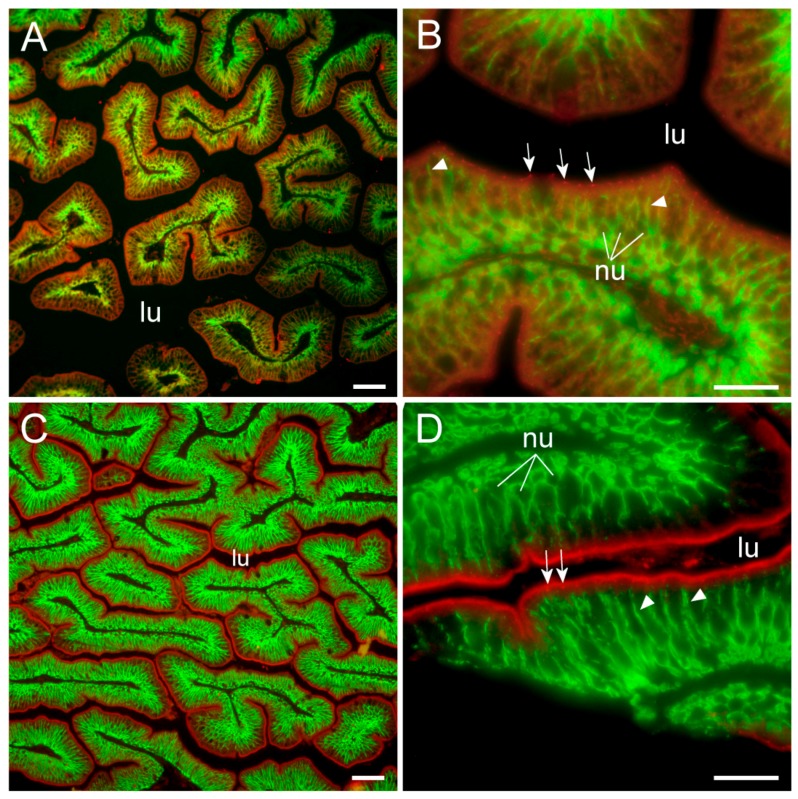
Immunofluorescence micrographs showing apical localization of Cldn15a (red, in **A** and **B**), and Cldn15b (red in **C** and **D**) and basolateral localization of the Na^+^,K^+^-ATPase alpha subunit (green) in FW-acclimated medaka middle intestine. (**A**) and (**C**) are at 200× magnification, (**B**) and (**D**) are at 1000× magnification. lu = lumen, nu = nuclei; in (**B**) and (**C**) arrows point to Cldn “hot spots” in the tight junction zones; arrowheads point to lateral membranes. Size bars indicate 50 μm (**A**, **C**) or 20 μm (**B**, **D**).

**Figure 7:**
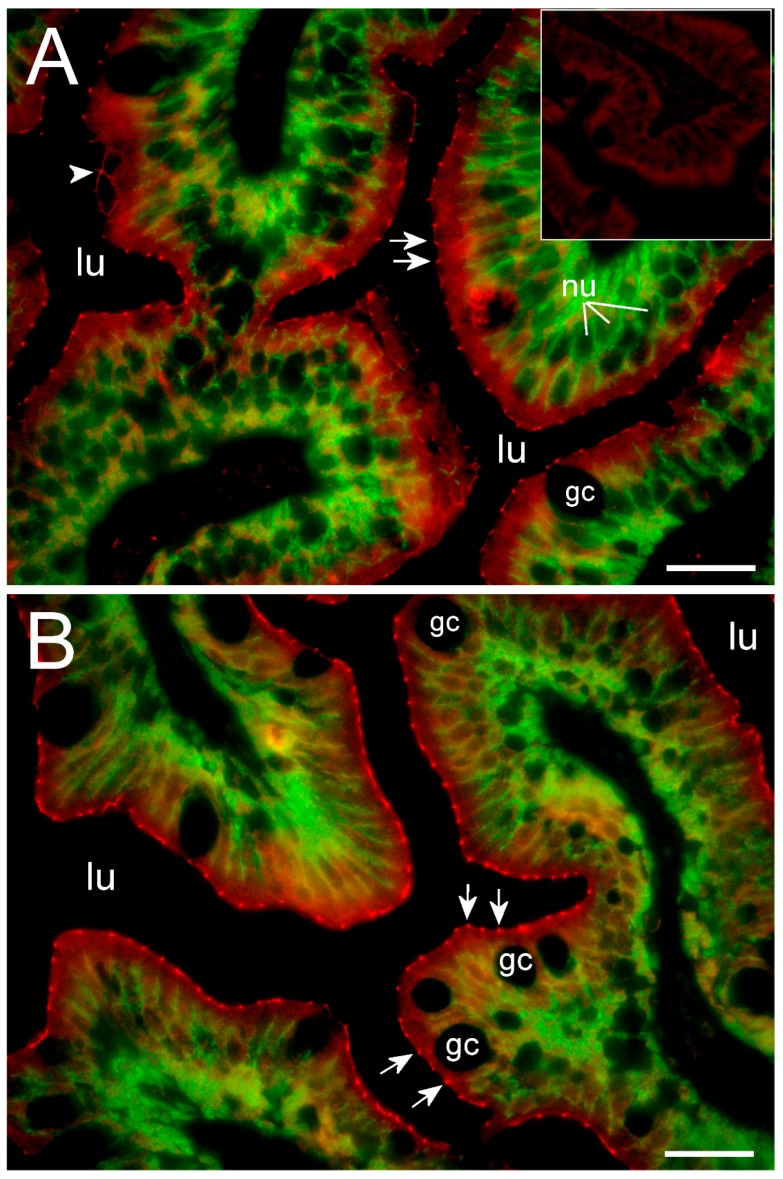
Immunofluorescence micrographs showing apical localization of Cldn15a (red in **A**) and Cldn15b (red in **B**) and basolateral localization of the Na^+^,K^+^-ATPase alpha subunit (green) in SW-acclimated medaka middle intestine. The insert in the upper left corner shows control without primary antibodies. Images are at 1000× magnification. lu = lumen, gc = goblet cell, nu = nuclei; arrows point to “hot spots” in the tight junction zones; Size bars indicate 20 μm.

Cldn15a and Cldn15b showed similar localization in the intestinal epithelium. Immunoreactivity was confined to the apical area of enterocytes with distinct “hot spots” in the apical junction area between enterocytes (Figure 6 and Figure 7). At lower magnification, these hot spots were partly masked by the non-specific staining of the brush-border area at variable intensity (Figure 6A,C).

**Figure 8:**
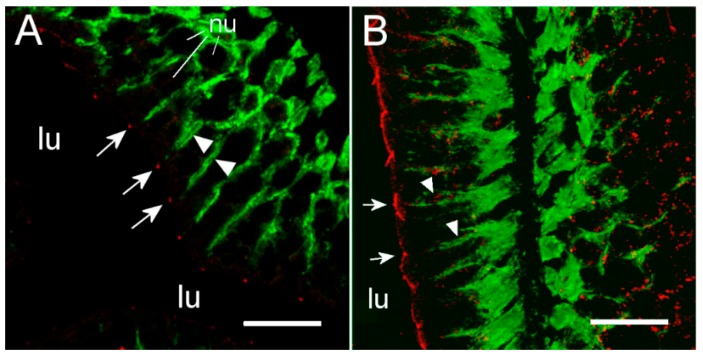
Confocal images showing apical localization of Cldn15a (red in **A**) and Cldn15b (red in **B**) and basolateral localization of Na^+^,K^+^-ATPase alpha subunit (green) in SW-acclimated medaka middle intestine. lu = lumen; arrows point to Cldn15 “hot spots” in the tight junction zones; arrowheads point to lateral membranes of enterocytes clearly separating the intercellular space. Size bars indicate 10 μm.

**Figure 9:**
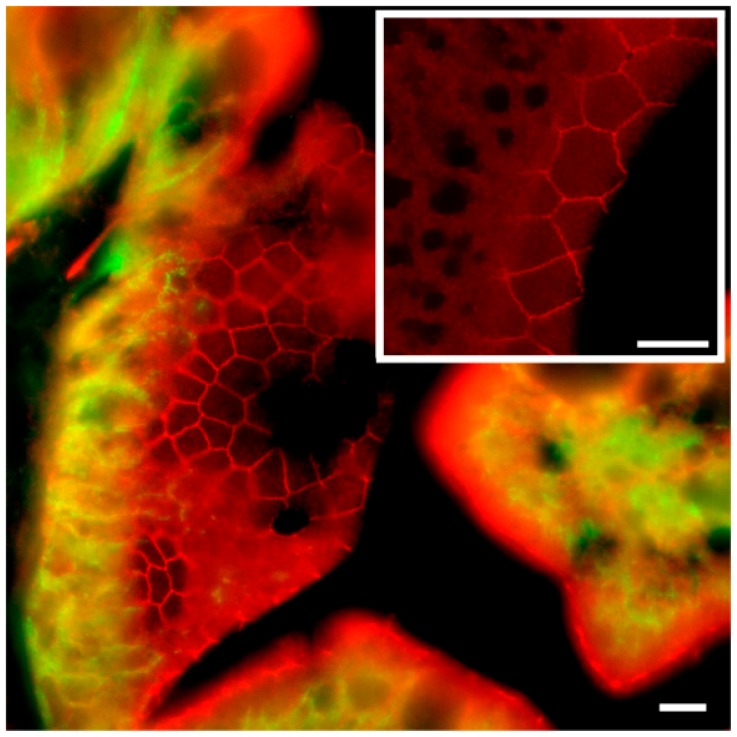
Confocal STED images showing the apical localization of Cldn15b (red) in SW-acclimated medaka middle intestine. Large image shows double staining with the anti-Na^+^,K^+^-ATPase alpha subunit (green). Na^+^,K^+^-ATPase is localized in basolateral membranes as shown in Figure 6, Figure 7 and Figure 8, and it is absent in the apical area, where the tight junctions are located. Thus, the mosaic-like pattern of Cldn15b is without green overlay. The subfigure shows a subsection of the apical area focusing on the tight junction area. Size bars indicate 5 μm.

However, these “hot spots” became particularly evident at higher magnification (Figure 6B,D, Figure 7) and when inspecting the tissues with confocal and STED microscopy, which has a much narrower z-plane focus (Figure 8 and Figure 9). Control incubation without primary Cldn15 antibodies showed a very faint general fluorescence without the distinct “hot spots” (insert in Figure 7A). Na^+^,K^+^-ATPase alpha subunit immunostaining revealed parallel lateral membranes, which were slightly spaced between neighboring cells, thus creating the lateral intercellular space (e.g., see Figure 6D, Figure 8A,B, marked with arrowheads). Near the basal borders, the membrane staining surrounded the nuclei, which appeared as circular dark “holes” in the images (marked “nu” in Figure 6, Figure 7 and Figure 8). The distinct Cldn staining was apical to the Na^+^,K^+^-ATPase staining, i.e., at the end of an axis extrapolated from the lateral membrane area. Thus, there was no co-localization of the two antibodies. This indicates that the two Cldn15 proteins are located in the tight junction zone.

Occasionally, the section plane was slightly tilted and therefore made it possible to obtain a zoomed view of the apical junction area just below the brush border zone (Figure 9). In these cases, confocal STED microscopy showed a beautiful polygonal staining revealing the three-dimensional junction zone surrounding the individual enterocytes. These polygons varied from simple tetragons to heptagons in shape, indicating enterocytes surrounded by four to seven neighboring cells.

**Figure 10:**
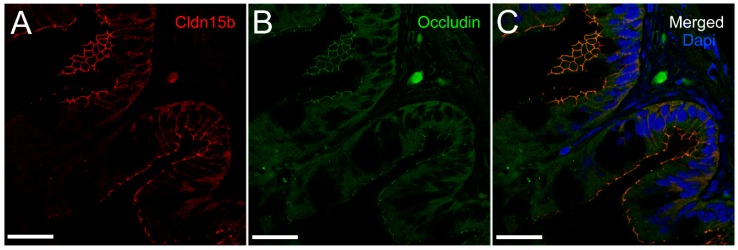
Confocal images showing Cldn15b (**A**, red), occludin (**B**, green) and co-localization (**C**, merged) in SW-acclimated medaka middle intestine. In (**C**), nuclei are stained blue with DAPI. Size bars indicate 20 μm.

We also performed a double labeling with Cldn15b and occludin antibodies (Figure 10). This revealed a complete co-localization of the two proteins, thus validating that Cldn15 is indeed localized in the tight junction between enterocytes.

## 3. Discussion

Japanese medaka can move between FW and SW while maintaining osmotic homeostasis. Based on our knowledge from several other teleosts, this requires high functional plasticity in e.g., the intestine, which in FW contributes to maintain ion balance and in SW switches to fluid absorption to compensate for dehydration [36]. Fluid absorption in fishes is driven by solute transport and is generally assumed to occur mainly through a transcellular pathway [2,7]. Accordingly, intestinal *aqp* expression is elevated during hyperosmotic exposure in order to develop the transcellular pathway [37,38]. This paradigm was challenged in previous studies, where we and others showed that in medaka *spp*., unlike in other species studied, intestinal *aqp*/Aqp expression is downregulated at both transcript and protein levels when fish are exposed to hyperosmotic conditions [8,39]. This paradox suggests that the paracellular pathway may be of higher importance, at least in the medaka. The recent report that the mammalian tight junction CLDN15 may create intestinal water channels [20] led us to investigate the role of the medaka orthologues in relation to fluid absorption. With limited knowledge about medaka drinking behavior and intestinal water handling, we set out by examining salinity effects on drinking behavior and water handling and then addressing the specific expression of *cldn15*. If involved in paracellular fluid absorption, our expectation was that *cldn15* expression would increase after SW exposure.

### 3.1. Drinking Rate and Intestinal Handling of Imbibed Water

After transition to SW, drinking rates and fluid absorption in the intestine increase in most examined fish species [36]. In order to understand intestinal function using the adult medaka model, we had to describe its drinking behavior and water handling, which was until now unknown. We demonstrated that the drinking rate was 5 μL/g/h and 10 μL/g/h in FW and SW medaka, respectively (Figure 1); thus, SW-transfer doubled oral water intake, which was presumably due to the need to compensate for osmotic water loss in the concentrated environment. This is similar to what has been observed in other euryhaline fish [35,40,41] including Japanese medaka larvae [42], and rates are comparable to other studies albeit on the high side [43]. Drinking rate is inversely related to body mass [43] and probably related to surface-to-volume ratio aspects, and most fishes examined up to now were larger fish (5–800 g). The medaka used in these experiments are small (0.4–0.6 g), and the smaller SW fish examined so far have comparable drinking rates (*Pholis gunnelus*, 2–10 g: 12 μL/g/h; *Aphanius dispar*, 0.4–1 g: 10 μL/g/h; see [43]). It is often assumed that FW fish should keep oral water intake to a minimum in order to not put excessive strain on the kidney in a hypotonic environment [36]. This is certainly the case in some FW teleosts (e.g., 0.4 μL/g/h in 0.1–2.5 g *Platichthys flesus* [44]). However, there are also reports of significant drinking in FW teleosts in the μL/g/h-range [41,45,46], and this also seems to be the case in FW medaka. We do not have any physiological explanation as to why FW drinking rates were so relatively high compared to most other reported studies. Feeding events could possibly be accompanied by the swallowing of small amounts of water, but normally, including the present study, fish are unfed during drinking rate measurements. Stress is another factor that may affect drinking and water turnover, but the fish were left undisturbed during the whole experiment, so we must assume that it is negligible. It has been speculated that drinking in FW may be a source of divalent ions such as Ca^2+^ [45], but the significance of this was rejected by Lin et al. [46] based on quantitative analyses in tilapia.

When analyzing the segmental distribution of 51-Cr-EDTA-traced water in the intestine (Figure 2), our data showed that in FW fish, 51-Cr-EDTA was for the most part located anteriorly in contrast with a more posterior accumulation in SW fish. This is in perfect agreement with Kaneko and Hasegawa’s [42] observations in medaka larvae, using a laser scanning technique to visualize intestinal water handling. This suggests that imbibed water in FW fish is taken up by osmosis in the anterior intestine were the tracers accumulate, and the volume regulatory problem associated must then be corrected by the kidney. While it is difficult to get reliable measurements of luminal fluid in small medaka, a study on FW tilapia showed that anterior, middle, and posterior luminal osmolality is close to plasma levels [47]. This corroborates an equilibration of the luminal fluid (FW) with plasma in the anterior intestine. In SW, after initial desalination in the esophagus, the luminal fluid osmolality in tilapia is similar all along the intestine and is higher than plasma osmolality [47]. Therefore, water uptake must rely on solute-driven transport into the lateral intercellular space in the anterior parts of the intestine [6] in combination with CaCO_3_ precipitation in the posterior end to increase the concentration of free water molecules [2,36]. Therefore, continuous water flow along the intestine means that 51-Cr-EDTA tends to accumulate in the posterior section in SW fish. The accumulation of the non-absorbable tracer, 99-Tc-DTPA, at the posterior end indirectly supports the progressive absorption of water. This was further supported by the SPECT/CT imaging, in which the progress of water movement in the intestine was visualized directly in live fish (Figure 3). In SW fish, CT scans further revealed mineral precipitation in the posterior intestine, suggesting bicarbonate secretion, which induces Mg- and Ca-carbonate precipitation that helps drive osmotic water transport across the intestinal epithelium [2].

### 3.2. Transcript Levels and Response to Salinity

The progress of SW acclimation was followed by transcript analyses of a few selected targets representing intestinal NaCl uptake (*nkcc2*), which is needed to establish solute-driven water absorption and a possible transcellular water uptake pathway (*aqp1a, aqp8ab*) (Figure 4 and Figure 5). As expected, there was a steep increase in *nkcc2* in SW, suggesting increased NaCl transport across the apical enterocytic brush border membrane. The data also confirmed the paradoxical drop in *aqp1a* and *aqp8ab* expression found in previous studies [*O. latipes*: 8; *O. dancena*: 39]. Thus, based on *aqp* dynamics, transcellular water transport is not supported in SW medaka; and it remains puzzling as to why *aqp* expression is kept higher in the FW condition. The time-course experiment showed that *cldn15b* was not significant affected by SW before 24 h and 168 h while the inhibitory effect on the two *aqps* was apparent at the 6 h and 24 h mark but not significant at the 168 h time point. Thus, while the dynamics of regulation are not straightforward, the overall inhibitory effect of SW on *cldn15b*, *aqp1a*, and *aqp8ab* observed previously was confirmed [8,34].

**Figure 11:**
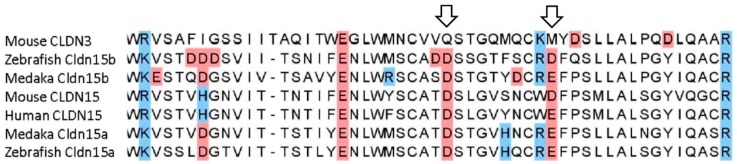
Alignment of the first extracellular loop of CLDN15 from human and mouse, and the orthologues from Japanese medaka and zebrafish shows that the residues critical to pore formation (D55 and D64) are found in both teleost Cldn15 paralogues. There are also differences from mammalian CLDN15; for example, both medaka Cldn15a and Cldn15b have an R63 residue and Cldn15a has an added H60. Amino acids are highlighted in red when acidic and in blue when basic. Arrows marks aspartic acids (D55 and D64), which are found to be important for cation and the water pore function of CLDN15 [11,12,15,16]. Mouse CLDN3 has been classified as a barrier protein and included as a reference. Sequences used: Human CLDN15: Acc. No. NP_001172009; Mouse CLDN15: Acc. No. NP_068365; Mouse CLDN3: NP_034032; Medaka Cldn15a: XP_004079873; Medaka Cldn15b: XP_004076514; Zebrafish Cldn15a: NP_956698; Zebrafish Cldn15b: NP_001035404.

Based on similarities to the mammalian CLDN15 shown in Figure 11, we hypothesized that the two medaka orthologs, *cldn15a* and *cldn15b*, may share functional properties in terms of forming cation and water pores and therefore may contribute to paracellular water absorption in SW medaka. In fishes (medaka [34]; salmon [31,32]; zebrafish [33]) and mammals [12], CLDN15 orthologs are expressed especially, but not exclusively, in the GI tract. In Atlantic salmon, SW acclimation was shown to induce elevated intestinal *cldn15a* mRNA expression [32], and a different study in the same species documented higher transepithelial resistance in SW than FW intestine measured *ex vivo* [48]. Taken together, this seems counterintuitive if teleost Cldn15 paralogs such as the mammalian ortholog form cation selective pores, and thereby theoretically should *decrease* epithelial resistance rather than increase it. We did not find any effect of salinity on *cldn15a* mRNA levels in medaka. However, Na^+^ and water fluxes through human CLDN15 was recently shown to inhibit each other [20], and it is possible that physiological significance depends on the local chemical conditions, which may be very different in FW and SW intestines. We found a roughly 100-fold decrease of the *cldn15b* paralog when fish are acclimated to SW, which based on an expected possible role in creating a water pore is somewhat surprising. The high FW expression level of this paralog suggests a specific role in the FW intestine, which may be related to Na^+^/glucose cotransport or K^+^ uptake from the diet. The interpretation of Cldn data is not straightforward, because Cldn15 may interact with other proteins and Cldn paralogs when co-expressed in enterocytes [25], and the properties and physiological significance may change depending on salinity and intestinal location. Therefore, the expression of other intestinal Cldns should be investigated in future studies.

### 3.3. Cldn15 Localization in the Intestinal Epithelium

To our knowledge, this is the first study showing enterocyte tight junction Cldn localization in a teleost fish. By using high-resolution fluorescence microscopy (Figure 6, Figure 7, Figure 8 and Figure 9), we were able to demonstrate that Cldn15a and Cldn15b showed similar localization in the intestinal epithelium regardless of salinity. The use of Na^+^,K^+^-ATPase immunostaining to visualize basolateral membranes showed that parallel lateral membranes were slightly spaced between neighboring cells, thus creating the lateral intercellular space possibly involved in solvent drag [7]. Discrete Cldn15 immunoreaction was seen apically to Na^+^,K^+^-ATPase immunoreaction, demarcating apical and basolateral membranes with no co-localization of the two antibodies. The antibodies gave some apparent non-specific staining of the brush border zone, which had variable intensity between sections. It is possible that this is created by non-specific adsorption to the mucus layer in this area. Nonetheless, the specific immunoreaction of both Cldn15 antibodies was restricted to a very narrow apical-most zone, which was below the brush border and in direct extension from membranes bordering the lateral intercellular space. In cellular cross-sections at high resolution, this appeared as an apical dot-like staining, and when viewed from above in a frontal section, the pattern appeared as a circumcellular polygonal pattern, which is characteristic of epithelial tight junctions. This became particularly evident at higher magnification when using high-resolution STED microscopy. The co-localization of Cldn15b with the tight junction marker occludin confirmed a role in control of the paracellular intestinal barrier. The localization is identical to that of CLDN15 throughout the mouse intestine [49] and that of occludin in the goldfish intestine [50]. Despite their classification as tight-junction proteins, several other intestinal CLDNs (e.g., CLDN-1, -3, -4, -5, and -7) are localized further away from the apical zone in lateral and basolateral membranes in mammals (see [25]). Based on the mRNA analyses, we expected to see a significant downregulation of Cldn15b after SW-exposure, but we did not find any significant change in the localization and immunoreactivity of neither Cldn15 paralog. There was a trend that the “hot spots” of Cldn staining appeared more intense in SW specimens, but it was not possible to quantify this (compare Figure 6B,D with Figure 7A,B). Unfortunately, the antibodies did not function for Western blots, and further quantification efforts are not possible at present. Thus, we conclude that the Cldn15-based apical tight junction component is resilient to changes in salinity, suggesting that it may contribute to paracellular fluid transport.

### 3.4. Conclusion and Perspectives

The drinking rate in FW medaka is quite high though still increasing when fish are challenged with hyperosmotic conditions. This suggests that the need for fluid absorption increases as dehydration threatens osmotic homeostasis. Several Aqp isoforms are expressed in the medaka intestine [8], but paradoxically, the most abundant forms (Aqp1a, Aqp8ab, and Aqp10 [8]) are significantly downregulated in SW, in parallel with the increased demand for fluid uptake. This led us to hypothesize that in medaka, the paracellular pathway may be more important when fish move into a hyperosmotic environment. The present data do not reject this hypothesis but do not provide strong support, either. Cldn15 paralogs make a significant constituent of the apical tight junction complex and may thereby create a paracellular water (and Na^+^) leak pathway. However, there are no signs that this is reinforced during SW acclimation.

The present study leaves behind a couple of questions: (1) What is the physiological significance of drinking in FW, and is this water really absorbed in the intestine? (2) What is the significance of uncoupled transcript and protein dynamics with regard to the Cldn15b paralog? We have attempted to analyze unidirectional water fluxes across isolated gut segments ex vivo using tritiated water as a tracer in an Ussing chamber setup, but so far, the data are inconclusive due to the fragility of the tissue. Future research in this area should pursue the functional aspects of water transport in this species by including vivo knock-down technology as well as analyses of luminal fluid chemical composition.

## 4. Materials and Methods

### 4.1. Fish and Rearing Conditions

The Japanese medaka (*Oryzias latipes*) used for this study came from two different sources. The fish (CAB strain) used for histological examinations, drinking rate, and drinking-related experiments were purchased from the UMS AMAGEN (Centre national de la recherche scientifique, Gif-sur-Yvette, France) and held in tanks with biofiltered FW or 30 ppt at 24–26 °C and exposed to a 12:12 light:dark photoperiod. These fish were generally fed four times per day with TetraMin^®^ flakes (Tetra GmbH, Melle, Germany), and the food was withheld 2 days before any experimentation. The experimental procedures were approved by the Danish Animal Experiments Inspectorate in accordance with the European convention for the protection of vertebrate animals used for experiments and other scientific purposes (#86/609/EØF). Long-term acclimated fish for transcriptional analysis were obtained from Aquatic Research Organisms, Inc. (Hampton, NH, USA; CAB strain) and held in biofiltered FW or 30 ppt SW and sampled after 1 month of acclimation. They were fed daily with TetraMin tropical flakes (Tetra, United Pet Group, Blacksburg, VA, USA), and food was withheld 2 days prior to any sampling. To investigate the early response to hyperosmotic environments, 10 female and 10 male FW-acclimated medaka were transferred to both sham FW conditions and SW (30 ppt; Instant Ocean, Spectrum Brands, Blacksburg, VA, USA; *n* = 40) and sampled after 6, 24, and 168 h (*n* = 6 per group). All handling and experimental procedures were approved by the Animal Care and Use Committee of the University of Arkansas (IACUC 17091).

### 4.2. Drinking Rate Measurements

A series of experiments was performed to estimate the rate of drinking in FW and SW-acclimated medaka. The gamma emitter 51-Cr-EDTA (PerkinElmer, NEZ147001MCNSA1, Waltham, MA, USA) was used as a non-absorbable marker for these experiments. The tracer (5 MBq) was added to the water (1 L of FW or 30 ppt SW), and the fish (*n* = 10–12) were then transferred to the experimental tank [35,40]. They were allowed to drink for 3 h, after which they were transferred to clean water (1 L) for 3 min and transferred to another tank with clean water (1 L) for 30 min. Then, the fish were anaesthetized in 100 mg/L MS-222 (Tricaine methanesulfonate) and killed by cervical dislocation. Before dissection, the fish were blotted by a paper towel and weighed to the nearest mg. The intestine was carefully ligatured at the anterior and posterior ends, removed from the body, and transferred to a 5 mL scintillation vial. The head was separated from the body with remaining organs and transferred to separate scintillation vials. All samples had 0.5 mL of distilled water added, and they were counted on a PerkinElmer 1480 WizardTM 3” Automatic gamma counter. The radioactivity of a 1.0 mL water sample was measured to estimate the specific radioactivity of the drinking water. Background radioactivity was counted on a 1.0 mL non-radioactive water sample. All samples were corrected for background, and the specific drinking rate (μL/g/h) was calculated as DR = sa/(bw*time), DR = drinking rate, sa = background-corrected specific activity (counts/minute); bw = body weight; time = time in radioactive water. Prior to these experiments, the accumulation of 51-Cr radioactivity was investigated and found to be linear in excess of 3 h.

### 4.3. Water Passage through the GI Tract

Medaka are agastric fish, meaning that the esophagus is directly connected to the anterior part of the tube-like intestine. In order to trace the passage of imbibed water in FW and SW-acclimated fish, two fish were allowed to drink for 3 h in water to which 51-Cr-EDTA had been added as described above. After this the fish were anaesthetized in MS-222 and killed by cervical dislocation, and the complete GI tract was ligatured at both ends and removed from the fish. Then, segments of 5–6 mm were ligatured and carefully dissected into scintillation vials to estimate the longitudinal distribution of radioactivity. The counting and calculations were done according to the above methodology, and the data were graphed in percent of total radioactivity as a function of longitudinal position.

### 4.4. Single Photon Emission Computed Tomography (SPECT)–Computed Tomography (CT) Scanning

In order to visualize the intestinal passage of imbibed water, a series of experiments was done in which fish were allowed to drink water with added non-absorbable marker 99-Tc-DTPA (Technetium-99mTc-diethylene-triamine-pentaacetic acid). This short-lived gamma emitter (T_1/2_ = 6.0067 h) is a widely used clinical radiophamaceutical for renal diagnosis and functioning. Subsequently, the fish were analyzed by SPECT-CT scanning. SPECT scanning is used for three-dimensional analysis of the radiochemical, while CT scanning creates a three-dimensional X-ray image, and when the two images are merged, a high-resolution image localizing the radiochemical to internal structures is obtained. All SPECT/CT scans were performed on a Siemens INVEON multimodality pre-clinical scanner (Siemens pre-clinical solutions, Knoxville, TN, USA).

Three fish were used for experiments in FW and SW, respectively, with imaging time-points at 1, 4, and 6 h for each group. For each salinity, two fish were transferred to a container with 100 mL water with the addition of 3.5 GBq Tc-99-DTPA, and one fish was transferred to a container with 100 mL of water with the addition of 5 GBq Tc-99-DTPA. Due to the short half-life of the 99-Tc isotope, a relatively high specific activity in the water is needed in order to obtain a good signal-to-noise ratio for visualization; thus, a higher activity was required for the late imaging group. All fish were allowed to drink in the labeled water for 1 h. Then, they were transferred to separate containers with 1 L of clean water for 5 min, followed by transfer to a second container with 1 L of clean water to rid the external surface for radioactivity. For each salinity, one fish was then euthanized in an overdose of MS-222 after a total of 1, 4, and 6 h after transfer to clean water and analyzed by SPECT-CT scanning in order to analyze the progressive movement of the imbibed isotope through the GI tract. After euthanasia, each fish was wrapped in plastic to avoid dehydration during the following imaging. The fish was placed in a lateral position on a dedicated SPECT/CT pre-clinical bed (25 mm).

CT scans were performed with the following settings; 360° rotation with 360 projections and 2 × 2 bin. The magnification was set at medium, yielding an isotropic pixel size of 40.00 µm and a trans-axial field view of 42 mm. The tube voltage was set to 80 kV, the current was 500 µA, and each projection was exposed for 1000 ms. CT scans were reconstructed using Feldkamp algorithm, with a Sheep–Logan filter and slight noise reduction. SPECT images were acquired using mouse high-resolution single pinhole collimators. A full 360° rotation with 60 projections and a fixed radius of 25 mm yielded a reconstructed 28 mm trans-axial field of view. A 20% energy window centered on the energy peak of 99mTc at 140 keV was used. Acquisition duration was set to 100 sec/projection. CT and SPECT images were co-registered using a transformation matrix and SPECT data was reconstructed using the Siemens MAP3D algorithm (matrix 128 × 128, 0.5 mm pixels, 16 iterations, and 6 subsets).

### 4.5. RNA Isolation, cDNA Synthesis, and qPCR

RNA isolation was conducted according to the manufacturer’s protocol (TRI Reagent^®^; Sigma Aldrich, St. Lois, MO, USA). All samples were homogenized using a Power Max 200 rotating knife homogenizer (Advanced Homogenizing System; Manufactured by PRO Scientific for Henry Troemner LLC, Thorofare, NJ, USA). First, 500 ng of total RNA was used for cDNA synthesis using the Applied Biosystems High Capacity cDNA Reverse Transcription kit (Thermo Fisher, Waltham, MA, USA). Used primers were previously validated and published in Bossus et al. [34] and Madsen et al. [6]. Elongation Factor 1 alpha (*ef1α*), beta actin (*βact*), and ribosomal protein L7 (*rpl7*) were analyzed as normalization genes in all experiments. Quantitative PCR was run on a Bio-Rad CFX96 platform (BioRad, Hercules, CA, USA) using SYBR^®^ Green JumpStart (Sigma Aldrich). qPCR cycling was conducted using the following protocol: a denaturation/activation step (94 °C) for 3 min, 40 cycles of a 15 s denaturation step (94 °C) followed by an annealing/elongation step for 60 s (60 °C), and finally a melting curve analysis at an interval of 5 s per degree from 55 to 94 °C. The absence of primer–dimer association was verified with no template controls (NTC). As an alternative to DNAse treatment, the absence of significant genomic DNA amplification was confirmed using total RNA samples instead of cDNA in a no reverse transcriptase control (NRT). Primer amplification efficiency was analyzed using a standard curve method with dilutions of the primers from 2 to 16 times. Amplification efficiency was used to calculate the relative copy numbers of the individual targets. Relative copy numbers were calculated by E_a_^ΔCt^, where Ct is the threshold cycle number and E_a_ is the amplification efficiency. Data were normalized to the geometric mean of the three normalization genes.

### 4.6. Immunofluorescence, Confocal, and Stimulated Emission Depletion (STED) Microscopy

The preparation of medaka intestines for immunofluorescence microscopy followed the procedures described previously [51]. Sections (0.5 cm) from the middle part of the intestines from medaka acclimated to FW and 30 ppt SW were sampled and fixed overnight in 4% buffered paraformaldehyde at 4 °C. After rinsing several times in 70% EtOH, the tissues were dehydrated overnight through a graded series of EtOH and xylene followed by embedding in 60 °C paraffin. Five-micron-thick transversal sections were cut on a microtome, and sections were placed on Superfrost plus (Gerhard Menzel GmbH, Braunschweig, Germany) slides before being dried overnight at 55 °C. Then, the tissue sections were hydrated through washes in xylene, 99%, 96%, and 70% EtOH and finally Na citrate (10 mM Na-citrate, pH 6.0). Antigen retrieval was performed by boiling the sections in the citrate solution for 5 min in a microwave oven and leaving them in the warm citrate solution for 30 min before being washed in 1× PBS (in mmol L^−1^: 137 NaCl, 2.7 KCl, 1.5 KH_2_PO_4_, 4.3 Na_2_HPO_4_, pH 7.3). Then, representative sections were blocked by incubation in 2% goat serum and 2% bovine serum albumin in 1× PBS for 1 h at room temperature. This was followed by dual labeling with a cocktail of an affinity purified polyclonal rabbit antibody against medaka Cldn15a or Cldn15b, respectively, in combination with the monoclonal mouse α5 antibody, which recognizes the alpha-subunit of the Na^+^,K^+^-ATPase in all vertebrates (The Developmental Studies Hybridoma Bank developed under auspices of the National Institute of Child Health Development and maintained by The University of Iowa, Department of Biological Sciences, Iowa City, IA, USA). In a separate experiment, sections were dual-labeled with Cldn15b and an occludin mouse monoclonal antibody (Invitrogen, product # 33-1500) in order to verify localization in the tight junction zone. Primary antibodies were diluted in 2% goat serum and 2% bovine serum albumin in PBS and incubated overnight at 4 °C. The polyclonal Cldn antibodies were custom-made in rabbits by Genscript (Piscataway, NJ, USA) against the following epitopes near the C-termini: Japanese medaka Cldn15a PAPTRSVVASTYGR, GenBank accession XP_004079873.1; Japanese medaka Cldn15b SHAAPSNYDRNAYV, GenBank accession XP_004076514.1). They were used at the concentrations 0.5 μg/mL (Cldn15a), 0.6 μg/mL (Cldn15b), and 5 μg/mL (occludin). The α5 antibody was used at 0.2 μg/mL.

For immunofluorescence and confocal microscopy, the following secondary antibodies were used for visualization: Alexa Flour^®^ 568 Donkey Anti-Rabbit IgG (H+L) at 1 ug/mL and Oregon Green^®^ 488 Goat Anti-Mouse IgG (H+L) at 2ug/mL (Invitrogen^TM^ Molecular ProbesTM, Carlsbad, CA, USA). The incubation time was 1 h at 37 °C for the secondary antibody. Then, sections were washed repeatedly in PBS, and coverslips were mounted using ProLong Gold antifade reagent (Invitrogen).

For STED microscopy, we used higher Cldn antibody concentrations: 0.7 μg/mL (Cldn15a) and 0.9 μg/mL (Cldn15b). The secondary antibodies used for STED were goat-anti-rabbit Abberior^®^ STAR 488 and goat-anti-mouse Abberior^®^ STAR 440SX (Sigma-Aldrich) at 1:200 and 1:1000 dilution, respectively. Negative control incubations with 2% BSA in PBS instead of primary antibodies were made routinely. The fluorescence was inspected on a Leica HC microscope (Manheim, Germany) and pictures of representative areas were captured using a Leica DC200 camera. Confocal images were taken on a Zeiss LSM510 META confocal microscope (CarlZeiss, Oberkochen, Germany) using a 63× objective with oil immersion. STED images were recorded using a Leica TSC SP8 STED setup. The excitation was done at 500 nm using a white light laser for Abberior^®^ STAR 488 and at 458 using an Argon laser for Abberior^®^ STAR 440SXP. The depletion laser (STED laser) was a 592 nm CW for both channels. The emission was recorded at 510–560 nm using the gated hybrid detector (0.3 ns) in counting mode for the Abberior^®^ STAR 488 and at 500–550 nm using the non-gated hybrid detector in counting mode for the Abberior^®^ STAR 440SXP. The images were cross-talk corrected and deconvoluted using Huygens™ (Hilversum, Netherland). The deconvolution was done to further increase the resolution of the images and decrease the background.

### 4.7. Statistical Analyses

All data analysis was conducted using GraphPad Prism 8.0 software (San Diego, CA, USA). Data from the salinity transfer experiments were analyzed using Bonferroni adjusted two-tailed Student’s t-test in experiments with two groups and two-way ANOVA followed by Bonferroni’s multiple comparisons test of time-matched groups in experiments with more groups. Drinking rates were analyzed using two-tailed Student’s t-test. When required, data were log or square root transformed to meet the ANOVA assumption of homogeneity of variances as tested with Bartlett’s test. Significant differences were accepted when *p* < 0.05.

## Abbreviations

ANOVA	Analysis of variance
AQP	Aquaporin
CLDN	Claudin
CT	Computed tomography
DR	Drinking rate
DTPA	Diethylenetriamine penta-acetic acid
EDTA	2,2′,2″,2‴-(Ethane-1,2-diyldinitrilo)-tetraacetic acid
FW	Fresh water
GI	Gastrointestinal tract
NKCC	Sodium-potassium-chloride-cotransporter
SEM	Standard error of the mean
STED	Stimulated Emission Depletion
SPECT	Single photon emission computed tomography
SW	Seawater
PPT	Parts per thousand
